# Immune profiling of anti-SSA–positive at-risk individuals reveals increased type I IFN activity preceding lymphocyte abnormalities in Sjögren’s disease

**DOI:** 10.3389/fimmu.2026.1723672

**Published:** 2026-09-28

**Authors:** Jacob Ritter, Ana-Luisa Stefanski, Arman Aue, Hector Rincon-Arevalo, Luca Rapino, Ali Necmi Eren, Yidan Chen, Eduard Nitschke, Van Duc Dang, Annika Wiedemann, Eva Schrezenmeier, Andreia C. Lino, Franziska Szelinski, Thomas Dörner

**Affiliations:** 1 Department of Rheumatology and Clinical Immunology, Charité – Universitätsmedizin Berlin, Corporate Member of Freie Universität Berlin and Humboldt-Universität zu Berlin, Berlin, Germany; 2 German Rheumatism Research Center (Deutsches Rheuma-Foschungszentrum (DRFZ)), a Leibniz Institute, Berlin, Germany; 3 Berlin Institute of Health at Charité – Universitätsmedizin Berlin, Berlin Institute of Health (BIH) Academy, Berlin, Germany; 4 Department of Endocrinology and Metabolic Diseases, Charité-Universitätsmedizin Berlin, corporate Member of Freie Universität Berlin, Humboldt-Universität zu Berlin, Berlin, Germany; 5 Department of Nephrology and Intensive Care, Charité – Universitätsmedizin Berlin, Corporate Member of Freie Universität Berlin and Humboldt-Universität zu Berlin, Berlin, Germany; 6 Grupo de Inmunología Celular e Inmunogenética, Facultad de Medicina, Instituto de Investigaciones Médicas, Universidad de Antioquia (UdeA), Medellín, Colombia; 7 Reumatologia, Dipartimento di Scienze Cliniche Internistiche, Anestesiologiche Cardiovascolari, Sapienza University, Rome, Italy

**Keywords:** anti-SSA, autoimmunity, B cells, interferon, Sjögren’s disease

## Abstract

**Objectives:**

Identifying immune features that distinguish asymptomatic autoantibody-positive at-risk individuals from patients with established autoimmune disease is key to understanding the evolution from autoreactivity to established autoimmune disease. This study investigated immune characteristics of anti-SSA–positive at-risk individuals, including pregnant women, compared with patients with Sjögren’s disease (SjD) and healthy donors (HDs).

**Methods:**

Peripheral blood samples were obtained from asymptomatic anti-SSA–positive women (at-risk individuals; n = 7, including two ongoing pregnancies with congenital heart block [CHB] and one woman with a prior CHB pregnancy), patients with SjD (n = 10), and age- and sex-matched HDs (n = 21; median age 35.1 years; 100% female). T- and B-cell subsets and IFN signaling molecules, including STAT1, phosphorylated STAT1 (pSTAT1), STAT2, IRF1, and IRF9, were analyzed by flow cytometry. In addition, circulating IFN subtypes were quantified in available plasma samples using the NULISA platform.

**Results:**

At-risk individuals showed lower ANA and rheumatoid factor titers and lacked anti-SSB autoantibodies compared with patients with SjD. At-risk individuals had B-cell distributions similar to HDs, whereas patients with SjD showed a typical increase in naïve B cells and reduced pre-switched and switched memory B-cell frequencies, together with increased CD8+ T-cell frequencies and a non-significant trend toward reduced double-negative T-cell frequencies. At-risk individuals also showed significantly increased IFN activity, reflected by Siglec-1 (CD169) expression on CD14+ monocytes, compared with HDs, while levels tended to be lower than in patients with SjD. Two of three women with a current or previous CHB pregnancy had the highest Siglec-1 levels within the at-risk group. Cytokine profiling revealed a stage-associated IFN pattern, with IFN-α1 and IFN-ω already increased in at-risk individuals, whereas IFN-γ, IFN-β1, and type III IFNs showed a trend toward higher levels in established SjD. Intracellular STAT1 expression was elevated across CD19+ B-cell subsets in at-risk individuals compared with HDs, but was generally less pronounced than in SjD. IRF9 was also increased in B-cell subsets from at-risk individuals and patients with SjD compared with HDs. Similar findings were observed in CD3+ T-cell subsets, where STAT1 and IRF9 expression were elevated in at-risk individuals compared with HDs and were largely comparable to levels observed in patients with SjD.

**Conclusions:**

Our data suggest that increased type I IFN activity and IFN-associated signaling in T- and B-cell subsets are already present in anti-SSA–positive at-risk individuals. In contrast, altered B- and T-cell homeostasis was restricted to established SjD, indicating progression from autoreactivity toward overt autoimmune disease.

## 1 Introduction

Our understanding of the chronological development from health to loss of tolerance and ultimately to clinically overt Sjögren’s disease (SjD) remains limited. Similar to other autoantibody-mediated autoimmune diseases, such as systemic lupus erythematosus (SLE) and rheumatoid arthritis (RA) (1, 2), SjD is preceded by a prolonged subclinical phase in which loss of tolerance is reflected by the early appearance of autoantibodies, including antinuclear antibodies (ANAs), rheumatoid factor (RF), and/or anti-SSA autoantibodies, which can be detected years to decades before disease onset (1, 3). However, ANAs can be detected in up to 25% of the general population, and only a minority of ANA-positive individuals progress to autoimmune disease. Anti-SSA autoantibodies are characteristic of SjD but may also occur in other autoimmune diseases and in asymptomatic individuals (1, 4–6). Recent studies have improved our understanding of individuals at risk of SLE and have identified the IFN-system and metabolic dysregulation as early cellular changes in this risk population (7, 8). Comparable studies in SjD remain limited.

Women carrying anti-SSA autoantibodies, including asymptomatic anti-SSA–positive at-risk individuals, have an increased risk of pregnancy complications, particularly the rare but clinically relevant risk of congenital heart block (CHB). Data from a large French registry showed that 49% of previously asymptomatic mothers of children with CHB developed an autoimmune disease within 11 years, most commonly SjD or SLE without severe organ manifestations (9). Thus, mothers of children with CHB who do not initially fulfill criteria for autoimmune disease represent a clinically relevant population for studying how autoreactivity evolves into overt SjD or related autoimmune diseases (10).

In addition to anti-SSA autoantibodies, elevated Siglec-1 expression on CD14+ monocytes, a surrogate marker of type I IFN activity, has been identified as a risk factor for CHB development in anti-SSA–positive pregnancies (11). SjD is also characterized by increased IFN activity and IFN gene expression in peripheral blood and affected tissues (12, 13). We recently described activation of the IFN signaling pathway in T- and B-cell subsets from patients with SjD (12). This activity was linked to serological autoimmunity, particularly hypergammaglobulinemia, ANA titers, and anti-SSA/SSB autoantibodies (12). However, although Siglec-1 expression on CD14+ monocytes indicate a type I IFN signature in asymptomatic at-risk individuals, intracellular IFN signaling in T and B cells in this cohort remains insufficiently characterized.

In addition to IFN activity, increased B- and T-cell activation is considered a feature of SjD pathology, encompassing autoreactive B cells, autoantibody production, and an increased risk of B-cell lymphoma (14–17). Altered B-cell subset distributions have been reported in SjD, including expansion of naïve B cells and reduction of pre-switched and switched memory B cells in peripheral blood (14). The reduced frequency of memory B cells in peripheral blood may reflect their preferential recruitment to and retention within inflamed salivary glands (14, 18). Recent single-cell and spatial transcriptomic studies have demonstrated that B cells, including memory B-cell populations, accumulate and clonally expand within inflamed salivary gland tissue, indicating active local immune responses and further supporting this hypothesis (19).

However, knowledge of B-cell pathology and B-cell distribution in asymptomatic anti-SSA–positive at-risk individuals remain limited. Taken together, while the roles of IFN activity and B cells have been studied in SjD, less is known about asymptomatic anti-SSA–positive at-risk individuals and which cellular or molecular profiles distinguish this stage from established SjD.

In this study, we combined comprehensive flow cytometric immune phenotyping of T- and B-cell subsets with intracellular analysis of IFN pathway molecules in age- and sex-matched HDs, asymptomatic anti-SSA–positive women (at-risk individuals, including three women with current or previous CHB pregnancies), and patients with SjD. At-risk individuals showed increased IFN pathway molecules across several immune cell subsets, although generally at lower magnitude than patients with SjD. However, only patients with SjD showed significant alterations in B-cell subset frequencies, increased CD8+ T cells, and a trend toward reduced DN T cells, whereas at-risk women displayed B-cell profiles resembling those of HDs.

Our results suggest that increased type I IFN activity represents an early event in anti-SSA–positive autoreactivity, whereas broader cytokine involvement and B- and T-cell abnormalities may occur later during progression to established SjD.

## 2 Materials and methods

### 2.1 Study participants and cohort definition

We included peripheral blood samples from 10 patients with Sjögren’s disease (SjD), 7 asymptomatic anti-SSA autoantibody-positive women (at-risk individuals), and 21 healthy donors (HDs) from our outpatient unit at the Department of Rheumatology and Clinical Immunology, Charité – Universitätsmedizin Berlin. Demographic and clinical information for the cohorts is summarized in **Table 1**; **Supplementary Table 1**, and **Figure 1A**.

**Table 1:** Demographic data of the healthy donors, at-risk women and Sjögren’s disease patients.

	Healthy donors (HDs)	At-risk individuals	Sjögren´s disease patients (SjD)
Individuals *(n)*	21	7	10
Age, *mean (range) in years*	33 (25–45)	34 (27–41)	38.1 (31–43)
female, *n (%)*	21 (100%)	7 (100%)	10 (100%)
ESSDAI, *mean, (range)*	-	0	2.8 (0-6)
autoantibodies:*n (%)*			
ANA titer >1:160 *(median)*	-	7 (1:846)	10 (1:3744)
Anti-SSA positive	-	6 (86%)	9 (90%)
Anti-SSB positive	-	0 (0%)	7 (70%)
Anti-SSA and Anti-SSB positive	-	0 (0%)	7 (70%)
RF IgA U/mL (median, IQR)	-	93.38; (n=7)	253.27; (n=10)
RF IgM U/mL	-	64.04; (n=7)	213.44; (n=10)
Hypergammaglobulinemia %	-	0.0; (n=none)	28.22; (n=5)
pregnant at sampling, *n*	1	4	3
week at sampling	-	38, 33, 31, 22	11, 18, 19
CHB presence at sampling, *n*	-	2	0
Prior complications (abort or CHB) *n*	-	1x CHB, 4x abort	3x abort
medication (dosage) at sampling:			
Hydroxychloroquine, *n (mean dosage)*	-	5 (220 mg)	9 (255.55 mg)
Prednisolon, *n (mean dosage)*	-	2 (3 mg)	3 (10 mg)

Demographics and laboratory data of the 21 HDs, 7 at-risk women and 10 SjD patients enrolled.

ESSDAI, EULAR Sjögren’s syndrome disease activity index; ANA, antinuclear antibody; RF, rheumatoid factor; CHB, congenital heart block.

**Figure 1:**
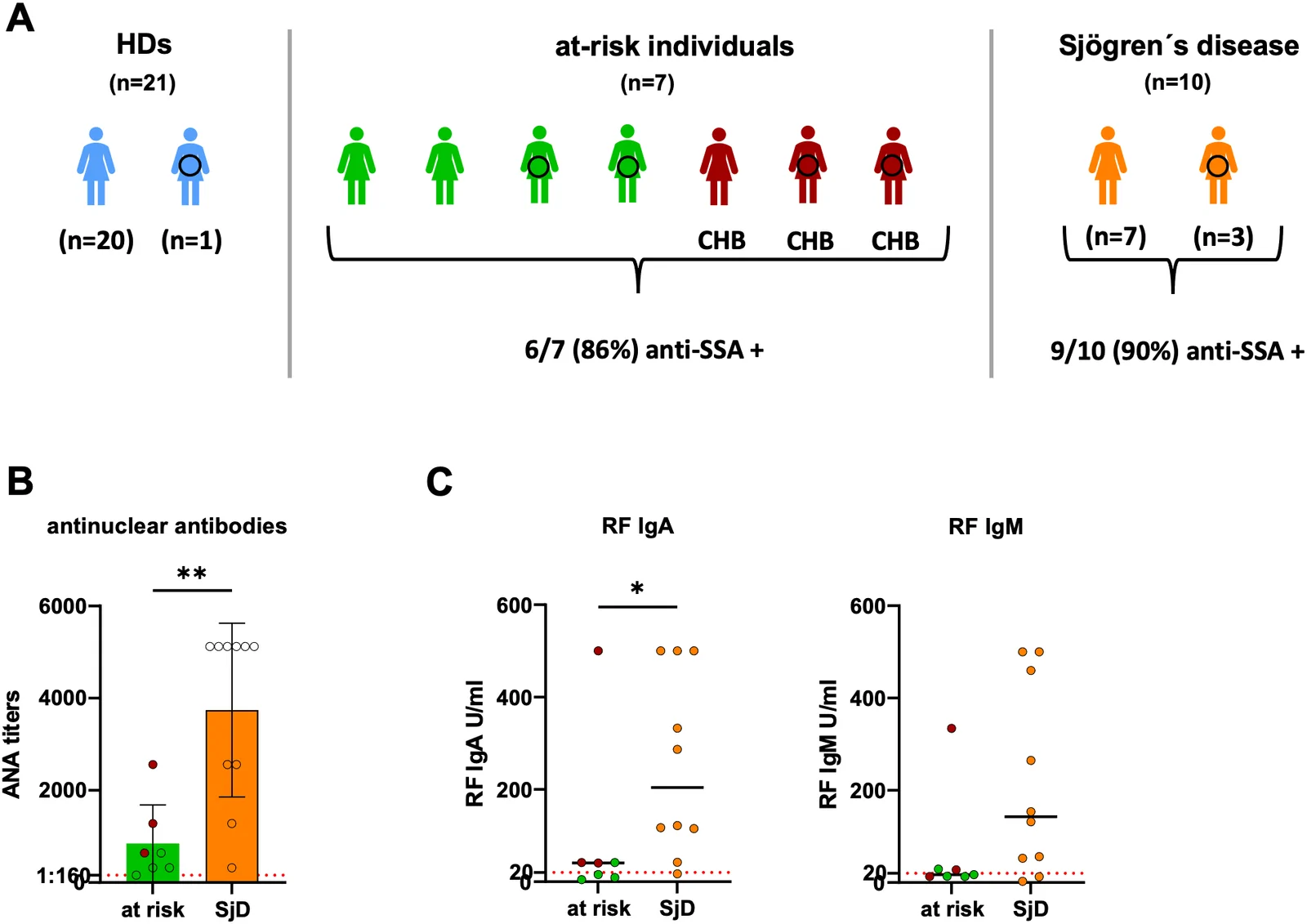
Serological differences between at-risk women and SjD patients. **(A)** Visualization of the recruited patient cohorts. Levels of **(B)** antinuclear antibody (ANA) titers and **(C)** rheumatoid factors (RF) IgA and IgM in U/ml between at-risk women and SjD patients. HDs in blue, n=21; 7 at-risk women without CHB complicated pregnancy (n=4 in green) and with CHB complicated pregnancy (n=3 in red); SjD patients in orange, n=10. Circles presented pregnancy during sampling. Scatter plots represent means ± SD; statistical significance was determined using two-tailed MWU *p ≤ 0.05, **p ≤ 0.01, ***p ≤ 0.001, ****p ≤ 0.0001. Normal ranges (dashed line) are shown.

At-risk individuals were defined as asymptomatic women who did not fulfill criteria for an autoimmune disease but had current or previous anti-SSA autoantibody positivity. These women are considered at risk for autoimmune complications such as CHB during pregnancy and/or subsequent progression to overt autoimmune disease. Four of seven at-risk individuals were pregnant at sampling. Three of seven at-risk individuals had CHB-related complications, including two ongoing pregnancies with CHB and one prior CHB pregnancy. Five at-risk individuals received hydroxychloroquine and two women received prednisolone as primary or secondary prophylaxis in women with a history of CHB or at risk of CHB, aiming to reduce maternal type I IFN activity and the risk of CHB occurrence or recurrence.

The SjD patients fulfilled the ACR/EULAR Sjögren´s disease criteria (20) and the average disease activity index for the SjD patients (European Sjögren disease activity index; ESSDAI) was 2.8 (21). One SjD patient suffered from malignant B cell lymphoma in the past. Two patients had arthritis involvement during sampling and one SjD patient was suffering from interstitial nephritis. The majority of SjD patients reported dry eyes, dry mouth and fatigue. Of note, none of the at-risk individuals reported SjD associated symptoms. Three out of ten SjD patients were pregnant during sampling. There was no CHB complication in this cohort.

Most patients with SjD were treated with hydroxychloroquine (HCQ; average dose 255 mg/day) and/or low-dose prednisolone (average 3mg/d). Whenever possible, serological data were collected during the outpatient visit, and disease activity was assessed using the ESSDAI score. All at-risk individuals, patients with SjD, and HDs were sex-matched (100% female), with a median age of 35.1 years.

Participants provided written informed consent, and the study was approved by the ethics committee of Charité – Universitätsmedizin Berlin (ethical approval number EA4/051/17) in accordance with the Declaration of Helsinki.

### 2.2 Laboratory findings for correlation analysis

Autoantibodies, including antinuclear antibody (ANA) titers, anti-SSA autoantibodies, anti-SSB autoantibodies, rheumatoid factor (RF) IgA and IgM, and immunoglobulin G (IgG), were assessed by the routine diagnostic laboratory testing.

### 2.3 Intracellular staining

Peripheral blood samples (EDTA anticoagulated; BD Vacutainer system, BD Diagnostics, Franklin Lakes, NJ, USA) were collected from all cohorts. Intracellular staining of Signal Transducer and Activator of Transcription 1 (STAT1), phosphorylated STAT1 (pSTAT1 Tyr701), STAT2, interferon regulatory factor 1 (IRF1), and IRF9 in T- and B-cell subsets was performed as previously described (13, 22). Erythrolysis and fixation of peripheral whole blood were carried out immediately after collection using BD Phosflow Lyse/Fix Buffer (BD Biosciences, San Jose, CA, USA; 10 min, 37 °C), according to the manufacturer’s instructions. After two washing steps, permeabilization was performed using BD Perm Buffer II (BD Biosciences, San Jose, CA, USA; 12 h, −20 °C), followed by overnight storage at −20 °C. The next day, cells were washed twice and resuspended in BD Brilliant Buffer (BD Horizon, San Jose, CA, USA). Cells were stained with antibodies after incubation with FcR blocking reagent (Miltenyi Biotec, Bergisch Gladbach, NRW, Germany). Further information regarding fluorochromes and volumes is provided in **Supplementary Table 2**. For analysis of IRF9 and STAT2, cells were incubated for 30 min with either an unconjugated rabbit IRF9 antibody (Thermo Fisher; isotype rabbit IgG; clone 14H9L22; 1:100) or an unconjugated rabbit STAT2 antibody (Cell Signaling; isotype rabbit IgG; clone D9J7L). STAT2 analyses were performed in samples from patients with SjD (n = 4) and at-risk women (n = 4). IRF9 analyses were performed in samples from patients with SjD (n = 9) and at-risk women (n = 4).

After washing, cells were incubated with a *donkey anti-rabbit IgG secondary antibody* (AF488, Jackson Immuno Research, isotype donkey anti-rabbit IgG, polyclonal, 1:100). Flow cytometric analysis was performed using a *BD FACS LSR Fortessa X-20* (BD Biosciences, Franklin Lakes, NJ, USA). The median fluorescence intensity (MFI) of various markers was measured. As a quality control, daily *Cytometer Setup and Tracking beads* (CST beads, BD Biosciences, Franklin Lakes, NJ, USA) and *Rainbow Calibration Particles* (BD Biosciences, Franklin Lakes, NJ, USA) were employed. Additionally, at least one healthy control sample was included in each analysis for comparison. Flow cytometry data were analyzed using *FACSDiva software* (Becton Dickinson, Franklin Lakes, NJ, USA) and *FlowJo* (version 10, TreeStar, Ashland, OR, USA).

### 2.4 Surface staining of Siglec-1 on CD14^+^ monocytes

Siglec-1 (CD169) expression on CD14^+^ monocytes, serving as a marker for the type I IFN signature, was measured by flow cytometry as reported previously (22).

### 2.5 Identification of B and T cell subsets

Cells were identified by gating on lymphocytes and single cells before discrimination of CD19+ B cells and CD3+ T cells (gating strategy in **Figure 2A**; **Supplementary Figure 1A**). CD14^+^ monocytes were identified within the CD19^-^ CD3^-^ compartment.

**Figure 2:**
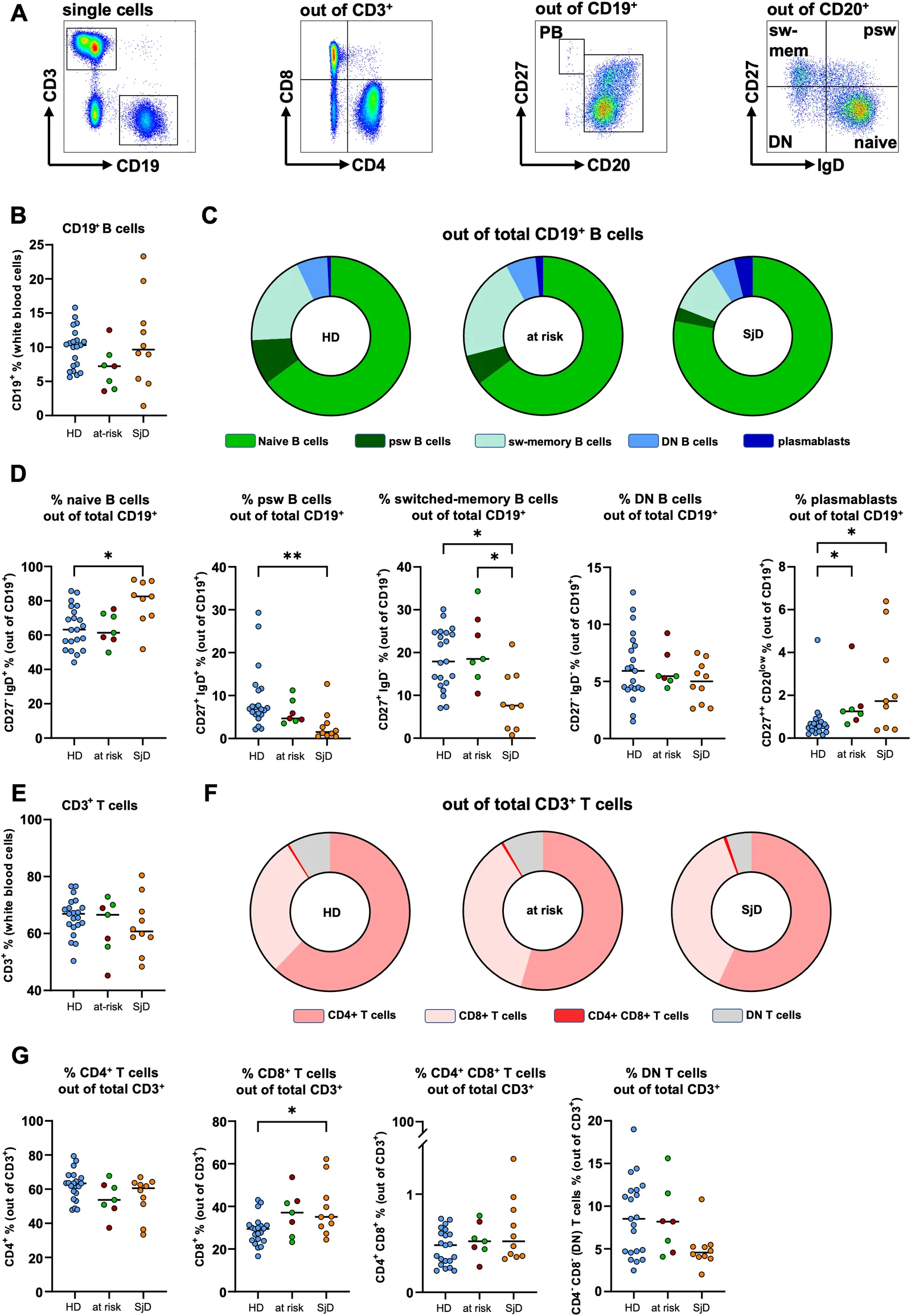
At-risk women show B-cell frequencies similar to HDs, whereas patients with SjD show disturbed B-cell distribution. **(A)** Representative gating strategy for the described cell subsets: based on CD3+ cells, CD4+ T cells (CD3+ CD4+), CD8+ T cells (CD3+ CD8+), CD4+ CD8+ T cells (CD3+ CD4+ CD8+), and CD4− CD8− double-negative T cells (CD3+ CD4− CD8−); based on CD19+ CD20+ cells, naïve B cells (IgD+ CD27−), pre-switched memory B cells (psw; IgD+ CD27+), switched memory B cells (mem; IgD− CD27+), double-negative B cells (DN; IgD− CD27−), and plasmablasts (PB; CD20low CD27++). **(B)** Total frequency of CD19+ B cells and **(C)** distribution of B-cell subsets within the CD19+ B-cell compartment across cohorts based on mean values. **(D)** Percentages of naïve, psw, memory, DN B cells, and plasmablasts among total CD19+ B cells in HDs, at-risk women, and patients with SjD. **(E)** Total frequency of CD3+ T cells and **(F)** distribution of T-cell subsets within the CD3+ T-cell compartment across cohorts based on mean values. **(G)** Percentages of CD4+, CD8+, CD4+ CD8+, and DN T cells among total CD3+ T cells in HDs, at-risk women, and patients with SjD. HDs in blue, n=21; in total 7 at-risk women, without CHB pregnancy (n=4 in green) and with CHB pregnancy (n=3 in red); SjD patients in orange n=10. Scatter plots represent means; statistical significance was determined using two-tailed MWU *p ≤ 0.05, **p ≤ 0.01, ***p ≤ 0.001, ****p ≤ 0.0001. **(C)** and **(F)** represented cell subset distribution out of 100% CD19^+^ B cells and CD3^+^ T cells.

B cell subsets were identified within the CD19^+^ compartment as follows:

conventional naïve (CD19^+^ CD27^-^ IgD^+^); pre-switched memory (psw; CD19^+^ CD27^+^ IgD^+^); conventional switched memory (mem; CD19^+^ CD27^+^ IgD^-^); double negative (DN; CD19^+^ CD27^-^ IgD^-^) B cells and plasmablasts (PB; CD19^+^ CD20^low^ CD27^++^).

T cell subsets were identified within the CD3^+^ compartment:

CD4^+^ T cells (CD3^+^ CD4^+^); CD8^+^ T cells (CD3^+^ CD8^+^); CD4^+^ CD8^+^ T cells (CD3^+^ CD4^+^ CD8^+^) and CD4^-^ CD8^-^ double negative (DN) T cells (CD3^+^ CD4^-^ CD8^-^).

### 2.6 Quantification of circulating interferon subtypes using the NULISA platform

A subcohort of the original study cohort was included in this analysis based on the availability of stored plasma samples. Plasma was collected and stored at −20 °C until analysis. Interferon (IFN) proteins were quantified using the NULISAseq™ Inflammation Panel AQ (Alamar Biosciences, Fremont, CA, USA), an ultrasensitive multiplex immunoassay platform based on NUcleic acid Linked Immuno-Sandwich Assay (NULISA™) technology combined with a next-generation sequencing (NGS) readout for protein detection and quantification, as previously published (23).

For the present study, analyses focused to the five interferon analytes included in the Inflammation Panel AQ (IFNα1/IFNα13, IFNα2, IFNβ1, IFNγ, and IFNω1). Absolute concentration values were used for all downstream statistical analyses. Measurements were performed according to the manufacturer’s recommended assay workflow and quality control procedures. An outlier analysis (Grubb´s test) was additionally performed to assess the robustness of the results.

### 2.7 Statistical analysis

Descriptive statistics were applied to summarize the demographic characteristics of the study participants. Graphical and statistical analyses were conducted using *GraphPad Prism* (version 9.00, GraphPad Software, La Jolla, CA, USA).

Statistical significance between two groups was assessed using the two-tailed Mann-Whitney U (MWU) test. For multiple comparisons, the Kruskal-Wallis test was applied.

Spearman correlation coefficient was calculated to detect possible correlations between different readouts.

Unless otherwise specified, scatter and bar plots display means ± SD. Statistical significances were considered for p-values less than 0.05 and are indicated in the figures as follows: * p < 0.05; ** p < 0.01; *** p < 0.001; **** p < 0.0001.

## 3 Results

### 3.1 Serological differences between at-risk individuals and patients with Sjögren’s disease

Anti-SSA autoantibodies are characteristic serological findings in SjD and are included in the objective domains of the ACR/EULAR classification criteria (20). However, they can also occur in asymptomatic individuals. In pregnant women, anti-SSA autoantibodies can cross the placenta and are considered a key driver of autoimmune CHB pathophysiology, independent of the mother’s clinical disease status. In this study, we included 21 HDs, 7 at-risk individuals (asymptomatic anti-SSA autoantibody-positive women, including 3 with CHB-related complications), and 10 patients with SjD (**Figure 1A**).

To balance the cohorts with respect to pregnancy status, pregnant women were included in each group. Four of seven at-risk individuals were pregnant during sampling; two of these had fetal CHB and were closely followed. One woman had a previous CHB pregnancy with successful delivery, and four women reported previous pregnancy losses of unknown cause. The SjD group included three pregnant patients without CHB complications (**Table 1**; **Figure 1A**). One HD was pregnant during sampling (**Table 1**).

The common characteristic of the at-risk cohort was anti-SSA autoantibody positivity in the absence of signs or symptoms of autoimmune disease. Six of seven at-risk individuals (86%) had anti-SSA autoantibodies at sampling. One woman had documented anti-SSA autoantibodies before recruitment into the study (**Table 1**; **Figure 1A**). No at-risk individual had anti-SSB autoantibodies. In the SjD cohort, 90% of patients had anti-SSA autoantibodies at sampling, and 70% additionally had anti-SSB autoantibodies (**Table 1**).

Anti-SSA autoantibodies belong to the group of antinuclear antibodies (ANAs). Therefore, we assessed ANA titers in patients with SjD and at-risk individuals. Patients with SjD had significantly higher ANA titers than at-risk individuals (**Figure 1B**). The average ANA titer was 1:846 in the at-risk cohort and 1:3744 in the SjD cohort (**Table 1**). The internal cut-off for ANA positivity was 1:160. Most at-risk individuals had low ANA titers, whereas patients with SjD showed a broader range of ANA titers (**Figure 1B**). Notably, at-risk individuals with CHB-related complications showed higher ANA titers within the at-risk group (**Figure 1B**).

Following these differences, we assessed additional serological abnormalities. Rheumatoid factors (RFs) were higher in patients with SjD than in at-risk individuals. These differences were observed for RF IgA (mean 253.27 U/mL in SjD vs. 93.38 U/mL in at-risk individuals) and RF IgM (mean 213.44 U/mL in SjD vs. 64.04 U/mL in at-risk individuals) (**Table 1**; **Figure 1C**). Overall, at-risk individuals showed low RF IgA and IgM levels, with only a few values above the threshold of 20 U/mL. Importantly, one woman with a previous CHB pregnancy showed high RF IgA (500 U/mL) and RF IgM (334.3 U/mL) levels within the at-risk cohort.

In summary, most at-risk individuals and patients with SjD were anti-SSA autoantibody positive. A notable difference was that patients with SjD showed higher serological activity, with co-occurrence of anti-SSB autoantibodies, higher RF IgA and IgM levels, and higher ANA titers compared with the at-risk group.

### 3.2 At-risk individuals showed a similar B-cell subset distribution to HDs, whereas plasmablast frequencies differed across the three cohorts

Although B cells play a crucial role in the pathophysiology of SjD and autoantibody-mediated autoimmune diseases, little is known about their frequency and distribution in at-risk individuals. Therefore, we first analyzed the frequency of CD19+ B-cell subsets (**Figure 2A**) and found no differences in the percentage of total CD19+ B cells among HDs, at-risk individuals, and patients with SjD (**Figure 2B**). We then performed a more detailed analysis of CD19+ B-cell subsets, including naïve, pre-switched memory, switched memory, double-negative B cells, and plasmablasts (see detailed phenotypic identification in **Figure 2A**). At-risk individuals exhibited a B-cell subset distribution similar to HDs, whereas patients with SjD displayed an abnormal B-cell subset distribution (**Figures 2C, D**), consistent with prior reports (24).

Specifically, patients with SjD showed a significantly increased frequency of naïve B cells compared with HDs, whereas at-risk individuals showed a distribution resembling that of HDs (**Figure 2D**). In parallel, pre-switched memory and switched memory B cells were significantly reduced in patients with SjD compared with HDs (**Figure 2D**). Switched memory B-cell frequencies also differed significantly between at-risk individuals and patients with SjD (**Figure 2D**). No differences were observed in the frequency of double-negative B cells among the three cohorts (**Figure 2D**). Importantly, plasmablast frequencies were elevated in both patients with SjD and at-risk individuals compared with HDs, while patients with SjD showed the highest increase (**Figure 2D**).

In conclusion, patients with SjD showed increased naïve B-cell frequencies and reduced pre-switched and switched memory B-cell frequencies, whereas at-risk individuals and HDs shared a similar naïve and memory B-cell distribution. Together with the elevated plasmablast frequencies observed in at-risk individuals and patients with SjD, these data suggest a graded pattern of B-cell activation, with early plasmablast expansion in at-risk individuals and more pronounced disturbance of naïve and memory B-cell compartments in established SjD.

### 3.3 Frequency of CD8^+^ T cells is increased in SjD patients compared to HDs

Next, we investigated differences in the CD3^+^ T cell population to determine whether disease-specific changes could be detected in T cell subsets. We gated for CD3^+^ T cells and further divided them into CD4^+^ and CD8^+^ T cells (**Figure 2A**). Initially, we assessed the percentage of total CD3^+^ T cells and found no differences among the groups (**Figure 2E**). Subsequently, we examined T cell subsets in greater detail. No differences were observed in the distribution of CD4^+^ T cells and double-positive (CD4^+^CD8^+^) T cells (**Figures 2F, G**).

However, the frequency of CD8+ T cells was significantly increased in patients with SjD compared with HDs (**Figure 2G**). At-risk individuals also showed slightly increased CD8+ T-cell frequencies, but this did not reach statistical significance (**Figure 2G**). In addition, patients with SjD showed a trend toward reduced double-negative (CD4−CD8−) T-cell frequencies compared with HDs and at-risk individuals, although this did not reach statistical significance (**Figure 2G**).

In summary, patients with SjD showed increased CD8+ T-cell frequencies and a non-significant trend toward reduced CD4−CD8− DN T-cell frequencies compared with HDs and at-risk individuals, paralleling the B-cell abnormalities described above.

### 3.4 Increased Siglec-1 (CD169) and circulating interferons in at-risk individuals and patients with SjD reflect stage-associated IFN activation

CD14+ monocytes are central to immune defense and IFN sensing. Siglec-1 (CD169) expression on CD14+ monocytes correlate with type I IFN activity and has been identified as a risk factor for CHB-related complications in anti-SSA–positive pregnant women as well as a biomarker in SjD (11, 22). Therefore, we compared Siglec-1 expression across the three groups.

Both at-risk individuals and patients with SjD showed significantly elevated Siglec-1 levels compared with HDs (**Figure 3A**). No significant difference was observed between at-risk individuals and patients with SjD. Notably, within the at-risk cohort, two of three women with current or previous CHB pregnancies had the highest Siglec-1 levels in this group (**Figure 3A**). In addition, the at-risk individuals with the highest Siglec-1 expression also had the highest ANA titers within the at-risk group and were positive for rheumatoid factors. This observation supports the concept that elevated autoantibody levels may contribute to type I IFN activation, potentially through immune complex–mediated stimulation.

**Figure 3:**
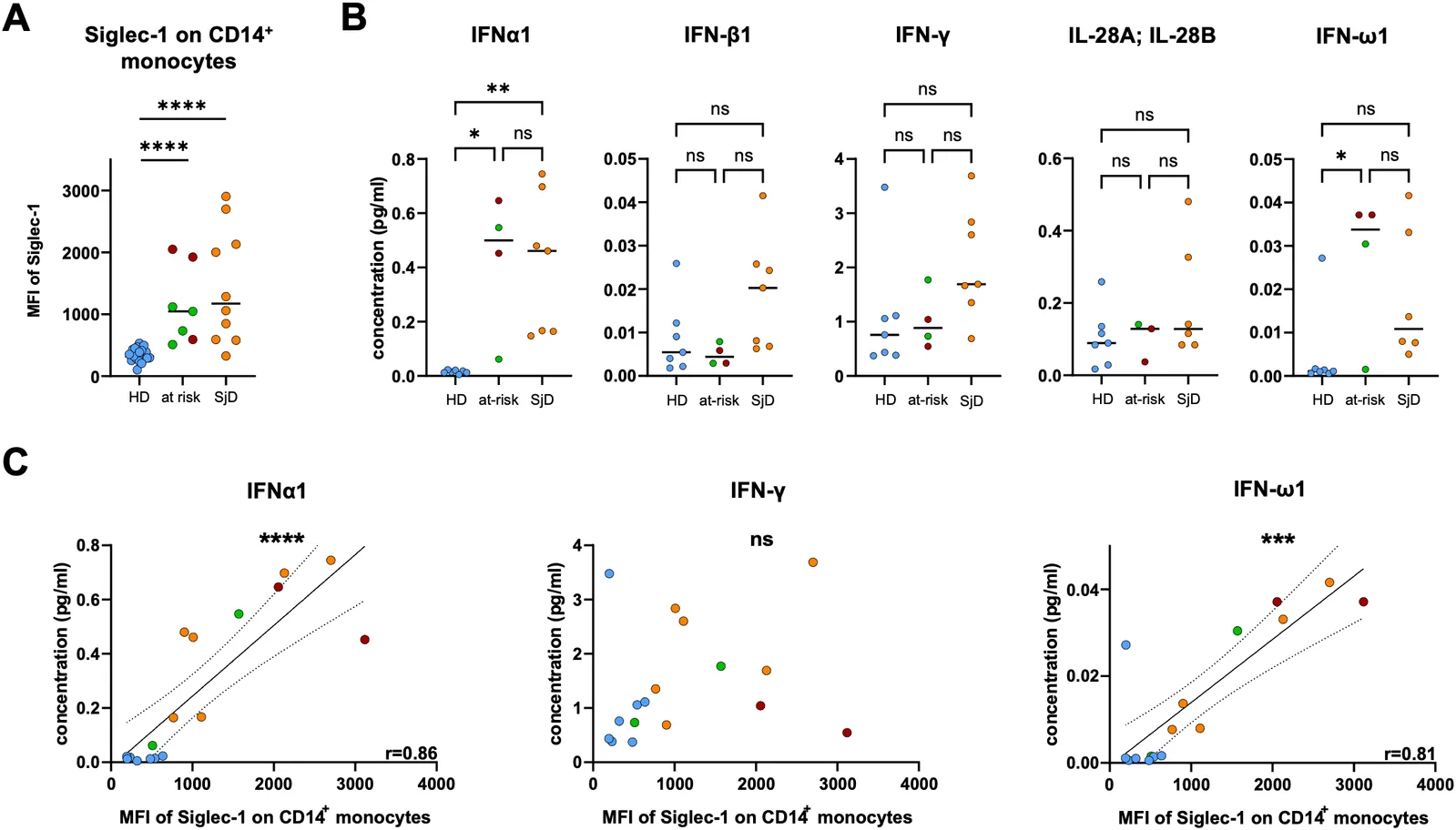
Increased Siglec-1 (CD169) and circulating interferons in at-risk individuals and patients with SjD reflect stage-associated IFN activation. **(A)** Median fluorescence intensity (MFI) of Siglec-1 (CD169) on CD14+ monocytes from HDs, at-risk women, and patients with SjD. **(B)** Absolute concentrations (pg/mL) of IFN-α1, IFN-β1, IFN-γ, IL-28A, IL-28B, and IFN-ω in HDs, at-risk women, and patients with SjD. **(C)** Correlations of IFN-α1, IFN-γ, and IFN-ω with Siglec-1 (CD169) expression on CD14+ monocytes in HDs, at-risk women, and patients with SjD. For **(B, C)** HDs in blue, n=7; at-risk women without CHB pregnancy (n=2 in green) and with CHB pregnancy (n=2 in red); SjD patients in orange n=7. Scatter plots represent means; statistical significance was determined using two-tailed MWU. Spearman’s rank test and coefficient (r) was calculated to identify correlations between the parameters. *p ≤ 0.05, **p ≤ 0.01, ***p ≤ 0.001, ****p ≤ 0.0001.

Next, we quantified circulating IFN subtypes in plasma. IFN-α1 was increased in both at-risk individuals and patients with SjD compared with HDs (**Figure 3B**). IFN-ω was also elevated in at-risk individuals compared with HDs. Notably, at-risk individuals with a clinically established CHB history or ongoing CHB pregnancy (highlighted in red) consistently displayed high IFN-α1 and IFN-ω levels within the at-risk group (**Figure 3B**). In contrast, IFN-γ, IFN-β1, IL-28A, and IL-28B (type III IFNs) showed a trend toward higher levels in patients with SjD compared with HDs and at-risk individuals (**Figure 3B**). An outlier analysis performed for the NULISA data confirmed the robustness of the primary findings (data not shown). These findings indicate a stage-associated IFN profile, with early type I IFN activity in at-risk individuals and broader IFN pathway involvement in established SjD.

IFN-α1 and IFN-ω correlated strongly with Siglec-1 expression on CD14+ monocytes (IFN-α1: r = 0.86, p = 0.0001; IFN-ω: r = 0.81, p = 0.001) (**Figure 3C**). This supports the use of Siglec-1 as a surrogate marker of circulating type I IFN activity. IFN-β1, IFN-γ, and type III IFNs did not correlate with Siglec-1 (**Supplementary Figure 2A**).

In summary, at-risk individuals and patients with SjD showed increased Siglec-1 expression, reflecting elevated type I IFN activity. In our study, two women with an ongoing CHB pregnancy showed strikingly increased Siglec-1 levels within the at-risk cohort, confirming earlier findings (11). We observed distinct IFN profiles at different stages of disease development, with IFN-α1 and IFN-ω already increased in at-risk individuals, whereas IFN-γ and IFN-β1 appeared to become more prominent in patients with established SjD.

Subsequently, we investigated IFN signaling components across cohorts and immune cell subsets. IFN signaling involves defined intracellular JAK/STAT pathways. After binding to IFN receptor domains, intracellular Janus kinases recruit and phosphorylate Signal Transducers and Activators of Transcription (STATs) (25). Phosphorylated STAT1 and STAT2 form a heterodimer together with interferon regulatory factor 9 (IRF9) and translocate as the ISGF3 complex to the nucleus, where the complex acts as a transcription factor (26) (**Figure 4A**). Therefore, we measured and compared key IFN signaling molecules, including STAT1, pSTAT1 (Tyr701), STAT2, and IRF9 in B-cell subsets from peripheral blood of HDs, at-risk women, and patients with SjD (**Figure 4A**; **Supplementary Figures 1B–D**).

**Figure 4:**
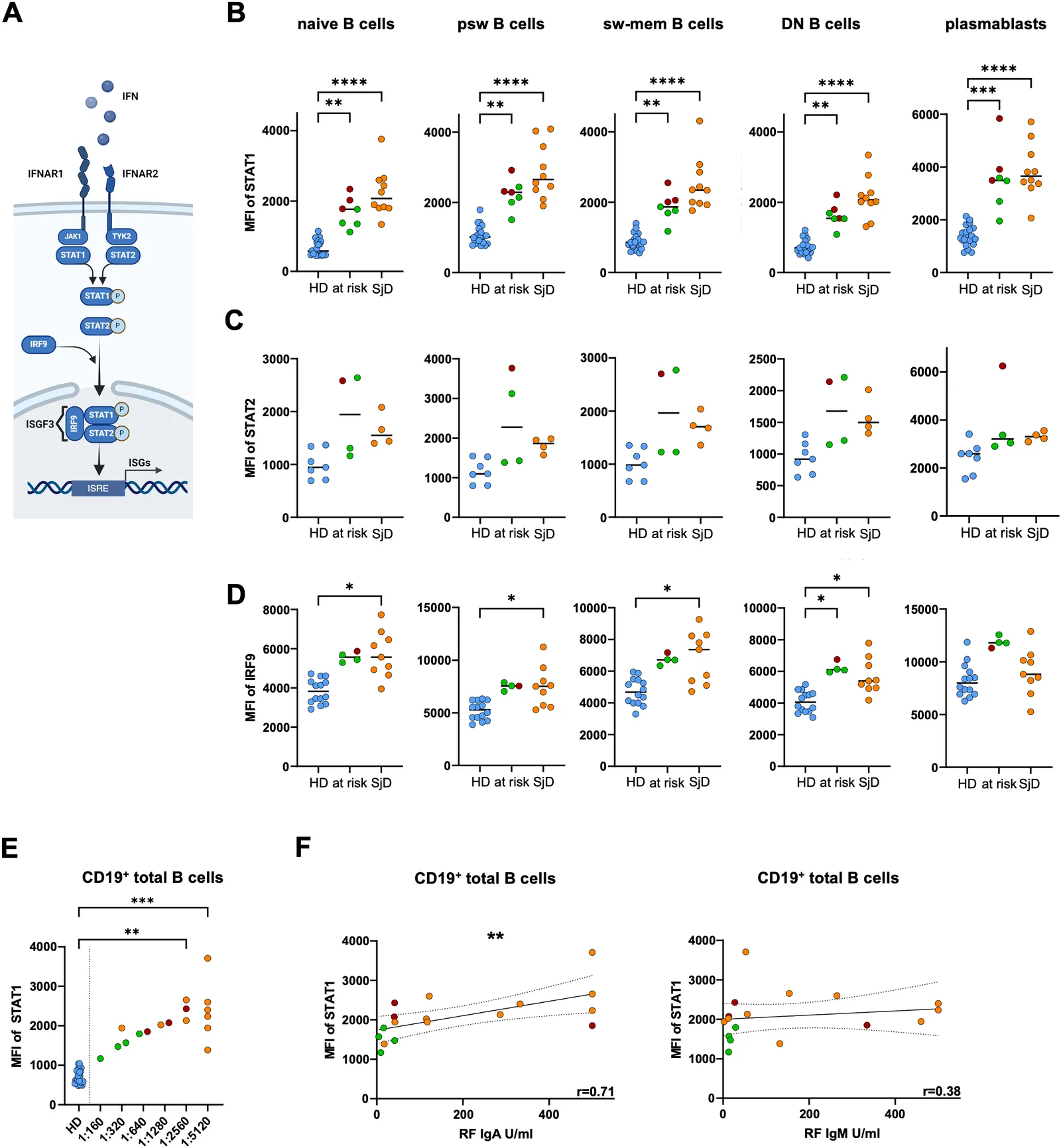
Increased STAT1 and IRF9 expression in B-cell subsets from at-risk women and patients with SjD. **(A)** Visualization of the JAK/STAT signaling pathway downstream of type I interferon. MFI of STAT1 **(B)**, STAT2 **(C)**, and IRF9 **(D)** in naïve, psw, memory, DN B cells, and plasmablasts from HDs, at-risk women, and patients with SjD. **(E)** MFI of STAT1 expressed by CD19+ B cells according to antinuclear antibody (ANA) titers. ANA titers were not determined in HDs, but STAT1 levels are shown as a reference. **(F)** MFI of STAT1 in CD19+ B cells correlates with IgA rheumatoid factor but not with IgM rheumatoid factor. HDs in blue, n=21; in total 7 at-risk women, without CHB pregnancy (n=4 in green) and with CHB pregnancy (n=3 in red); SjD patients in orange n=10. For STAT2: SjD and at-risk women n=4. For IRF9: SjD n=9 and at-risk women n=4. Graph **(B)** created with Biorender. Scatter plots represent means; statistical significance was determined using two-tailed MWU. Spearman’s rank test and coefficient **(r)** was calculated to identify correlations between the parameters. *p ≤ 0.05, **p ≤ 0.01, ***p ≤ 0.001, ****p ≤ 0.0001.

Across all investigated B-cell subsets, STAT1 expression was significantly increased in both at-risk individuals and patients with SjD compared with HDs (**Figure 4B**). Although no statistically significant differences were observed between at-risk individuals and patients with SjD, STAT1 levels tended to be higher in patients with SjD (**Figure 4B**). STAT1 expression was most prominently elevated in plasmablasts from at-risk individuals, particularly in those with a CHB pregnancy, who displayed the highest STAT1 levels within the at-risk cohort (**Figure 4B**).

Phosphorylated STAT1 was significantly increased in naïve and switched memory B cells from patients with SjD compared with HDs (**Supplementary Figure 2B**), whereas no differences were observed between at-risk individuals and HDs. For STAT2, we observed a trend toward increased levels in at-risk individuals and patients with SjD compared with HDs (**Figure 4C**). However, this association did not reach statistical significance, likely due to the small sample size. Overall, these observations are consistent with our previous findings of increased STAT2 expression in B-cell subsets from patients with SjD compared with HDs (12).

Patients with SjD showed significantly elevated IRF9 levels compared with HDs in naïve, pre-switched, switched memory, and DN B cells (**Figure 4D**). At-risk individuals showed a similar trend toward increased IRF9 expression across B-cell subsets compared with HDs, with a significant elevation in DN B cells (**Figure 4D**). No differences in IRF9 expression were observed in plasmablasts across the cohorts (**Figure 4D**).

In addition to IRF9, we measured IRF1 across cell subsets. IRF1 is induced by both type I and type II IFNs and regulates the expression of multiple ISGs, acting both as a downstream mediator of IFN signaling and as a regulator of basal immune gene expression (26). We did not observe differences in IRF1 expression in B-cell subsets across the three cohorts (**Supplementary Figure 2C**).

Given the established association between autoantibody production and type I IFN pathway activation, we investigated whether autoantibody levels were associated with IFN signaling activity in B cells. To this end, we analyzed the relationship between ANA titers, rheumatoid factors, and STAT1 expression in total CD19+ B cells. STAT1 levels in CD19+ B cells increased with higher ANA titers (**Figure 4E**), indicating a clear trend across the cohort. In addition, STAT1 levels in CD19+ B cells correlated with RF IgA, but not with RF IgM (**Figure 4F**).

In conclusion, at-risk individuals showed elevated intracellular IFN signaling molecules in B-cell subsets, with an overall profile more closely resembling patients with SjD than HDs, although STAT1 and pSTAT1 patterns suggest stronger downstream pathway activation in established SjD. In addition, ANA titers and RF IgA were associated with STAT1 expression in CD19+ B cells. These findings suggest that IFN-driven immune dysregulation may already contribute to the pathophysiology of at-risk individuals, while the lower level of serological activity and preserved B-cell homeostasis support a stepwise model of disease development.

### 3.5 T cells showed similar increased intracellular IFN signaling activity in at-risk individuals and SjD patients compared to healthy women

Next, we aimed to investigate potential alterations of IFN signaling in T cell subsets. This was particularly relevant since no significant differences were identified in the frequency of peripheral T cell subsets across our study cohorts.

To address this question, we performed intracellular flow cytometry analysis of IFN signaling pathway molecules in T-cell subsets (**Figure 2A**; **Figure 4A**). Across all investigated T-cell subsets, STAT1 MFI was significantly increased in both at-risk individuals and patients with SjD compared with HDs (**Figure 5A**). No differences were detected between the at-risk and SjD groups (**Figure 5A**).

**Figure 5:**
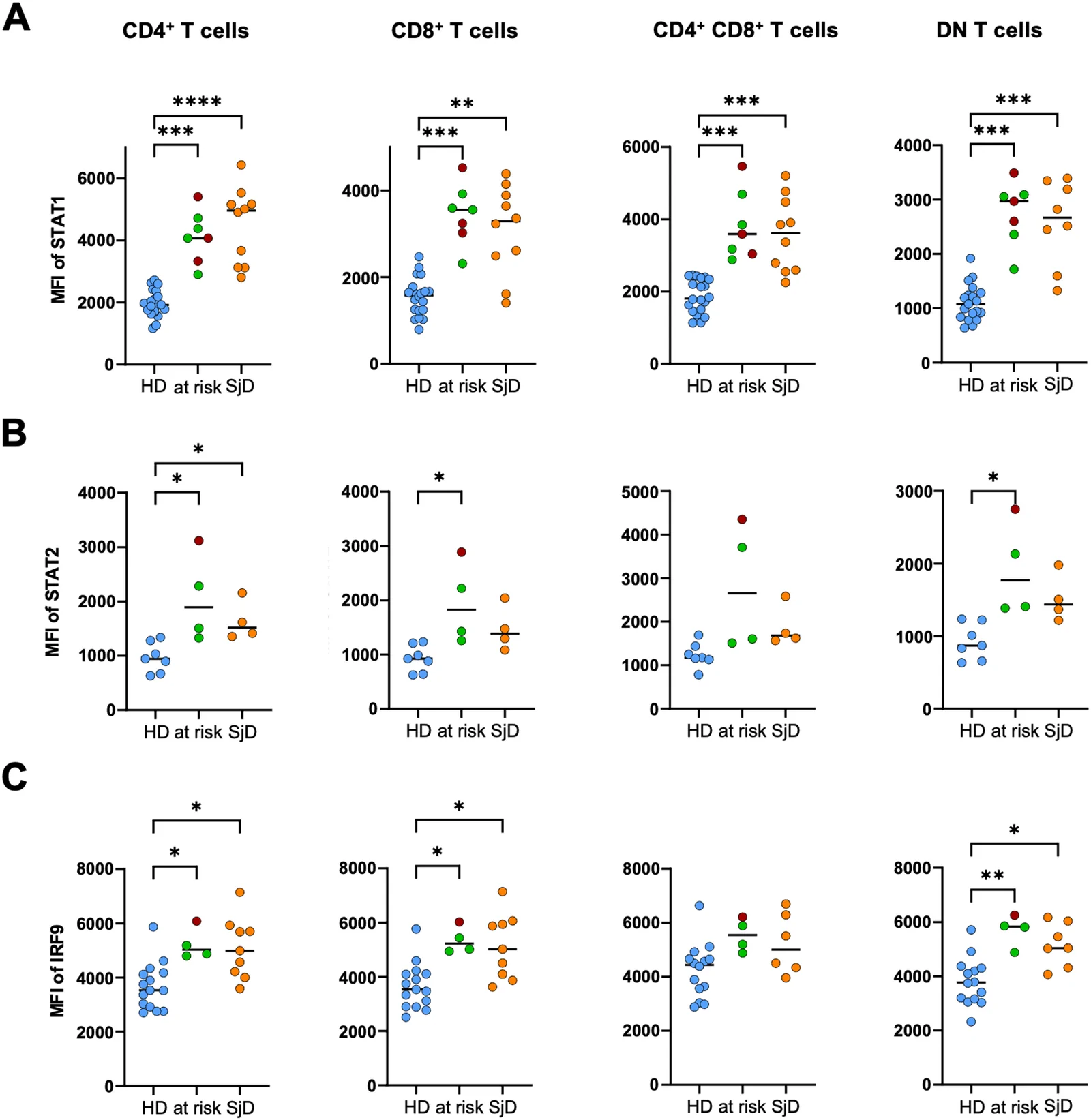
Increased STAT1 and IRF9 expression in T-cell subsets from at-risk women and patients with SjD. MFI of STAT1 **(A)**, STAT2 **(B)** and IRF9 **(C)** in CD4^+^, CD8^+^, CD4^+^ CD8^+^ and DN T cells from HDs, at-risk women and SjD patients. HDs in blue, n=21; in total 7 at-risk women, without CHB pregnancy (n=4 in green) and with CHB pregnancy (n=3 in red); SjD patients in orange n=10. For STAT2 and IRF9: SjD and at-risk women n=4. Scatter plots represent means; statistical significance was determined using two-tailed MWU **(A-C)**. *p ≤ 0.05, **p ≤ 0.01, ***p ≤ 0.001, ****p ≤ 0.0001.

We did not observe differences in pSTAT1 (Tyr701) expression in T-cell subsets across the three cohorts (**Supplementary Figure 3A**).

For STAT2, we observed statistically significant increases in at-risk individuals compared with HDs within CD4+, CD8+, and double-negative T-cell subsets (**Figure 5B**). In contrast, significant STAT2 elevations in patients with SjD compared with HDs were limited to CD4+ T cells (**Figure 5B**). However, conclusions regarding STAT2 must be interpreted with caution due to the small sample size. Similarly, IRF9 showed a pattern comparable to STAT2 within T cells (**Figure 5C**). IRF9 levels were significantly elevated in CD4+, CD8+, and DN T cells in at-risk individuals and patients with SjD compared with HDs (**Figure 5C**). Notably, within the at-risk cohort, a woman pregnant with a fetus diagnosed with CHB showed the highest STAT2 and IRF9 values across all investigated T-cell subsets (**Figures 5B, C**). We did not observe differences in IRF1 expression in T-cell subsets across the three cohorts (**Supplementary Figure 3B**).

In summary, STAT1 levels were consistently elevated in T-cell subsets from at-risk individuals and patients with SjD. These findings indicate IFN pathway activation in T cells, consistent with the corresponding findings in B cells. Together, these findings indicate that IFN-associated alterations affect both B- and T-cell compartments in at-risk individuals and patients with SjD, supporting early involvement of adaptive immunity and loss of tolerance before overt disease.

## 4 Discussion

This study explored clinical, serological, cellular, and molecular differences across three cohorts representing distinct stages along the autoimmune spectrum: patients with established SjD, asymptomatic anti-SSA–positive women at risk of developing autoimmune disease, and matched HDs. The at-risk cohort is particularly relevant because autoimmune CHB represents a severe pregnancy complication in anti-SSA–positive pregnant women. Nearly half of previously asymptomatic mothers of children with CHB develop SjD or related autoimmune diseases during long-term follow-up (9).

Thus, studying these individuals offers a unique opportunity to identify mechanisms involved in the transition from a healthy state through preclinical autoreactivity to overt SjD.

Here, we report that anti-SSA–positive at-risk women shared several serological features with patients with SjD, including anti-SSA autoantibodies and elevated type I IFN activity. However, they had lower ANA and RF titers and lacked anti-SSB autoantibodies. Importantly, our cohort of asymptomatic anti-SSA–positive at-risk women showed no glandular or extraglandular manifestations of autoimmune disease. These findings suggest that anti-SSA autoantibodies together with moderately elevated type I IFN activity are insufficient to induce clinical autoimmune disease by themselves and support the concept that additional pathogenic mechanisms or a “second hit” are required for disease manifestation.

The chronic IFN signature observed in at-risk women is consistent with previous studies of asymptomatic ANA-positive individuals at risk of systemic autoimmune diseases. Carter et al. reported transcriptional dysregulation characterized by enhanced IFN pathway activation in asymptomatic ANA+ individuals who may progress to SLE (7). Similarly, Hedlund et al. demonstrated elevated IFN-α levels and monocyte activation in anti-SSA/SSB-positive pregnancies and their newborns, suggesting sustained immune activation associated with autoantibody positivity (27). Furthermore, they demonstrated that fetal PBMCs can produce IFN-α upon exposure to anti-SSA autoantibody-positive plasma. Our findings of correlations between intracellular STAT1 expression and serological autoimmunity support the presence of a chronic type I IFN signature in at-risk individuals. Based on currently available studies, type I IFN activity appears to represent a shared immunological feature across several SjD subgroups rather than a phenomenon exclusively restricted to a single autoantibody subtype (19). This suggests that type I IFN pathway activation may be driven by common upstream mechanisms, including immune complex–mediated activation of innate immune responses. Whether IFN-α merely serves as a biomarker or plays an active pathogenic role remains to be delineated. Nevertheless, elevated IFN-stimulated gene expression is a well-described feature of SjD and has been implicated in disease pathogenesis (28–30).

Consistent with prior work, patients with SjD showed increased expression of IFN-related signaling molecules (12). At-risk women exhibited a similar intracellular IFN profile, although generally at lower magnitude. Specifically, both at-risk women and patients with SjD showed elevated STAT1 and IRF9 expression across T- and B-cell subsets, alongside increased Siglec-1 expression on CD14+ monocytes. These findings align with and expand previous work (11) by demonstrating that the IFN signature in at-risk women is not limited to monocytes but also involves adaptive immune compartments. Differential expression patterns of STAT1, STAT2, IRF1, and IRF9 likely reflect their distinct functional roles within IFN signaling pathways. STAT1 and IRF9 are closely linked to canonical IFN responses and positive feedback amplification, whereas STAT2 and IRF1 may be regulated differently depending on the dominant cytokine context and cell type (26, 31, 32). In particular, STAT1 can participate in both ISGF3-mediated type I IFN signaling and GAF-mediated IFN-γ signaling, while IRF1 may integrate IFN-γ–driven and basal inflammatory programs. Further functional studies are required to clarify the biological significance of these observations.

To characterize IFN activity in greater detail, we quantified circulating IFN subtypes in a subset of participants. The observed pattern suggests stage-dependent IFN involvement during disease development. Type I IFNs, particularly IFN-α1 and IFN-ω, were already increased in anti-SSA–positive at-risk individuals and may contribute to a permissive environment for loss of immune tolerance reflected i.e. by anti-SSA production. In contrast, broader engagement of additional IFN pathways, including IFN-γ and potentially IFN-β1 and type III IFNs, appeared more pronounced in established SjD. IFN-γ can promote B-cell activation indirectly through mediators such as BAFF and IRF1 (33), and has been implicated in salivary gland inflammation and BAFF production, a key driver of disturbed B-cell homeostasis and autoantibody generation in SjD (34, 35). The increased plasmablast frequencies observed in both at-risk individuals and patients with SjD may therefore reflect early B-cell activation in an IFN-rich, autoantibody-positive environment, whereas the additional cytokine signals detected in established SjD may contribute to progression toward more pronounced B-cell dysregulation. Together, these findings support a model in which early type I IFN activation precedes clinical disease, while subsequent engagement of additional inflammatory pathways contributes to overt autoimmunity.

Although the cohort size was limited, IFN-related abnormalities, including Siglec-1 expression, intracellular STAT1 and IRF9 levels, and circulating IFN subtypes, were generally more pronounced in patients with SjD than in at-risk individuals. These findings suggest progressive amplification of systemic IFN dysregulation during disease evolution rather than a binary distinction between health and disease.

B-cell dysregulation is a hallmark of SjD and contributes to the increased risk of B-cell non-Hodgkin lymphoma observed in these patients (15). In asymptomatic anti-SSA–positive at-risk women, detailed analyses of the B- and T-cell compartments remain limited. Previous studies have reported reduced frequencies of CD27+ memory B cells and expansion of naïve B cells in SjD, potentially reflecting retention and clonal expansion of memory B cells within inflamed salivary glands (14, 18). Recently, accumulation and clonal expansion of memory B cells within salivary gland tissue have been described (19). Our findings confirmed these characteristic alterations in patients with SjD. In contrast, at-risk women exhibited B-cell subset distributions largely comparable to HDs, without reduction of memory B cells or expansion of naïve B cells. T-cell populations were also largely preserved, although patients with SjD showed increased CD8+ T-cell frequencies and a non-significant trend toward reduced double-negative T cells. Thus, our data indicate that overt SjD is associated with peripheral B- and T-cell changes that may reflect chronic autoimmune processes and immune-cell redistribution to affected tissues, rather than impaired memory B-cell induction alone.

Our findings initially appear to contrast with those of Ivanchenko et al., who reported B-cell abnormalities in anti-SSA-positive women that are consistent with those observed in SjD (36). However, deeper analysis revealed that the majority of participants in the Ivanchenko study were diagnosed with SjD or SLE, respectively (36). Thus, their cohort aligns more closely with established systemic autoimmune conditions, rather than representing a unique feature of asymptomatic at-risk women.

All studies noted above (7, 9–11) highlight that the at-risk condition differs from HDs and may represent an intermediate state associated with increased risk of progression to overt autoimmune disease. One possible scenario is a “second-hit” or “multiple-hit” model, in which subsequent immune activation at a vulnerable time point promotes transition to overt SjD. Such events may include expansion of ANA reactivities, induction of anti-SSB autoantibodies (possibly by epitope spreading), increased RF reactivity, and broader IFN subtype production, with more intense involvement of chronic innate and adaptive immune abnormalities. Although the cellular sources of such intensified immune activation remain to be defined and are likely heterogeneous, similar pathogenic concepts have been proposed for SLE (1, 37) and RA (38).

This study has several limitations. First, the rarity of CHB resulted in a relatively small cohort size, limiting statistical power. Accordingly, these findings should be considered exploratory and require validation in larger, independent cohorts. Second, the cross-sectional design precludes conclusions regarding future disease progression. Longitudinal studies are necessary to determine whether the observed IFN abnormalities predict development of SjD or related autoimmune diseases. Third, further functional studies, especially stimulation experiments and IFNAR or JAK–STAT pathway blockade experiments, are required to strengthen the mechanistic interpretation of intracellular IFN signaling in this disease setting. Fourth, differences in pregnancy status between groups could theoretically influence immune parameters. However, pregnancy is generally associated with reduced IFN responses and lower B-cell numbers (39–41), whereas our at-risk cohort displayed increased IFN activity and preserved B-cell frequencies. Together with previous studies, these observations support the interpretation that the identified immune abnormalities are primarily driven by anti-SSA–associated autoimmunity rather than pregnancy itself (7, 27).

In conclusion, asymptomatic anti-SSA–positive at-risk women exhibit systemic type I IFN pathway activation resembling that observed in SjD, yet lack the characteristic B- and T-cell dysregulation and clinical manifestations of established disease. These findings suggest that type I IFN activation represents an early event in autoimmune disease development, whereas additional cytokine and cellular immune mechanisms are required for progression to overt SjD. Future studies should focus on identifying reliable predictors of CHB during pregnancy and elucidating how IFN activity and T- and B-cell abnormalities contribute to progression from an asymptomatic at-risk state to clinically established SjD.

## Data Availability

The raw data supporting the conclusions of this article will be made available by the authors, without undue reservation.

## Supplementary material

Supplementary Figure 1 **(A)** Representative gating strategy for the described cell subsets: naïve B cells (IgD+ CD27−), pre-switched memory B cells (psw; IgD+ CD27+), switched memory B cells (mem; IgD− CD27+), double-negative B cells (DN; IgD− CD27−), plasmablasts (PB; CD20low CD27++), CD4+ T cells (CD3+ CD4+), CD8+ T cells (CD3+ CD8+), CD4+ CD8+ T cells (CD3+ CD4+ CD8+), CD4− CD8− double-negative T cells (CD3+ CD4− CD8−), and CD14+ monocytes (CD3− CD19− CD14+). **(B)** Representative histogram for CD20+ B cells and median fluorescence intensity of STAT1, **(C)** STAT2, and **(D**) IRF9. **(E)** Representative histogram for CD3+ T cells and median fluorescence intensity of STAT1, **(F)** STAT2, and **(G)** IRF9. Representative histograms of molecules (blue/orange solid line), FMO (dashed line) and secondary antibody (light grey solid line) are shown.

Supplementary Figure 2 **(A)** Correlations of IFN-β1, IL-28A, and IL-28B with Siglec-1 (CD169) expression on CD14+ monocytes in HDs, at-risk women, and patients with SjD. **(B)** MFI of pSTAT1 (Tyr701) and **(C)** MFI of IRF1 in naïve, psw, switched memory, DN B cells, and plasmablasts from HDs, at-risk women, and patients with SjD. **(D)** STAT1 expression in B-cell subsets correlates with Siglec-1 levels on CD14+ monocytes in at-risk women. HDs in blue, n=21; in total 7 at-risk women, without CHB pregnancy (n=4 in green) and with CHB pregnancy (n=3 in red); SjD patients in orange n=10. Scatter plots represent means; statistical significance was determined using two-tailed MWU **(A, B)**. Spearman’s rank test and coefficient (r) was calculated to identify correlations between the parameters. *p ≤ 0.05, **p ≤ 0.01, ***p ≤ 0.001, ****p ≤ 0.0001.

Supplementary Figure 3 **(A)** MFI of pSTAT1 (Tyr701) and **(B)** MFI of IRF1 in CD4+, CD8+, CD4+ CD8+, and DN T cells from HDs, at-risk women, and patients with SjD. HDs in blue, n=21; in total 7 at-risk women, without CHB pregnancy (n=4 in green) and with CHB pregnancy (n=3 in red); SjD patients in orange n=10. Scatter plots represent means; statistical significance was determined using two-tailed MWU **(A, B)**. *p ≤ 0.05, **p ≤ 0.01, ***p ≤ 0.001, ****p ≤ 0.0001.

Supplementary Table 1 Demographics of all cohorts. Additional demographic, clinical data and laboratory data of the studied cohorts. ESSDAI , EULAR Sjögren’s Syndrome Disease Activity Index; PNS , peripheral nervous system; RF ,rheumatoid factor; CHB , congenital heart block.

Supplementary Table 2 antibody list. Summary of the applied monoclonal antibodies, analyzed surface markers incl. corresponding fluorochromes for flow cytometry (clones, volumes, isotypes, manufacturers, and catalog numbers).

## References

[1] Arbuckle MR McClain MT Rubertone MV Scofield RH Dennis GJ James JA . Development of autoantibodies before the clinical onset of systemic lupus erythematosus. N Engl J Med. (2003) 349:1526–33. doi: 10.1056/nejmoa021933 14561795

[2] Nielen MM van Schaardenburg D Reesink HW van de Stadt RJ van der Horst-Bruinsma IE de Koning MH . Specific autoantibodies precede the symptoms of rheumatoid arthritis: a study of serial measurements in blood donors. Arthritis Rheum. (2004) 50:380–6. doi: 10.1002/art.20018 14872479

[3] Theander E Jonsson R Sjöström B Brokstad K Olsson P Henriksson G . Prediction of sjögren's syndrome years before diagnosis and identification of patients with early onset and severe disease course by autoantibody profiling. Arthritis Rheumatol. (2015) 67:2427–36. doi: 10.1002/art.39214 26109563

[4] Tan EM Feltkamp TE Smolen JS Butcher B Dawkins R Fritzler MJ . Range of antinuclear antibodies in "healthy" individuals. Arthritis Rheum. (1997) 40:1601–11. doi: 10.1016/s0031-3025(16)38413-6 9324014

[5] Moutsopoulos HM Zerva LV . Anti-Ro (SSA)/La (SSB) antibodies and sjögren's syndrome. Clin Rheumatol. (1990) 9:123–30. doi: 10.1007/bf02205560 2203591

[6] Jia Y Luan S Huang S Zhang W Li M Xu T . Prevalence and clinical significance of anti-SSA antibody in the Chinese health screening population. Clin Exp Immunol. (2024) 218:169–76. doi: 10.1093/cei/uxae073 39136066 PMC11482497

[7] Carter LM Md Yusof MY Wigston Z Plant D Wenlock S Alase A . Blood RNA-sequencing across the continuum of ANA-positive autoimmunity reveals insights into initiating immunopathology. Ann Rheum Dis. (2024) 83:1322–34. doi: 10.1136/ard-2023-225349 38740438

[8] Md Yusof MY Psarras A El-Sherbiny YM Hensor EMA Dutton K Ul-Hassan S . Prediction of autoimmune connective tissue disease in an at-risk cohort: prognostic value of a novel two-score system for interferon status. Ann Rheum Dis. (2018) 77:1432–9. doi: 10.1136/annrheumdis-2018-213386 29929956 PMC6161671

[9] Miniaoui I Morel N Lévesque K Maltret A Driessen M Masseau A . Health outcomes of 215 mothers of children with autoimmune congenital heart block: analysis of the French Neonatal Lupus Syndrome Registry. J Rheumatol. (2022) 49:1124–30. doi: 10.3899/jrheum.210703 35501147

[10] Brito-Zerón P Pasoto SG Robles-Marhuenda A Mandl T Vissink A Armagan B . Autoimmune congenital heart block and primary sjögren's syndrome: characterisation and outcomes of 49 cases. Clin Exp Rheumatol. (2020) 38 Suppl 126:95–102. 33025893

[11] Lisney AR Szelinski F Reiter K Burmester GR Rose T Dörner T . High maternal expression of SIGLEC1 on monocytes as a surrogate marker of a type I interferon signature is a risk factor for the development of autoimmune congenital heart block. Ann Rheum Dis. (2017) 76:1476–80. doi: 10.1136/annrheumdis-2016-210927 28501799

[12] Ritter J Szelinski F Aue A Stefanski AL Rincon-Arevalo H Chen Y . Elevated unphosphorylated STAT1 and IRF9 in T and B cells of primary sjögren's syndrome: novel biomarkers for disease activity and subsets. J Autoimmun. (2024) 147:103243. doi: 10.1016/j.jaut.2024.103243 38788537

[13] Imgenberg-Kreuz J Sandling JK Björk A Nordlund J Kvarnström M Eloranta ML . Transcription profiling of peripheral B cells in antibody-positive primary sjögren's syndrome reveals upregulated expression of CX3CR1 and a type I and type II interferon signature. Scand J Immunol. (2018) 87:e12662. doi: 10.1111/sji.12662 29655283

[14] Hansen A Odendahl M Reiter K Jacobi AM Feist E Scholze J . Diminished peripheral blood memory B cells and accumulation of memory B cells in the salivary glands of patients with sjögren's syndrome. Arthritis Rheumatism. (2002) 46:2160–71. doi: 10.3138/ctr.145.3 12209521

[15] Nocturne G Mariette X . B cells in the pathogenesis of primary sjögren syndrome. Nat Rev Rheumatol. (2018) 14:133–45. doi: 10.1038/nrrheum.2018.1 29416129

[16] Broeren MGA Wang JJ Balzaretti G Groenen P van Schaik BDC Chataway T . Proteogenomic analysis of the autoreactive B cell repertoire in blood and tissues of patients with sjögren's syndrome. Ann Rheum Dis. (2022). doi: 10.1136/annrheumdis-2021-221604 35144926 PMC8995816

[17] Mauro D Lin X Pontarini E Wehr P Guggino G Tang Y . CD8(+) tissue-resident memory T cells are expanded in primary sjögren's disease and can be therapeutically targeted by CD103 blockade. Ann Rheum Dis. (2024) 83:1345–57. doi: 10.1136/ard-2023-225069 38777379

[18] Hansen A Daridon C Dörner T . What do we know about memory B cells in primary sjögren’s syndrome? Autoimmun Rev. (2010) 9:600–3. doi: 10.1016/j.autrev.2010.05.005 20452465

[19] Inamo J Takeshita M Suzuki K Tsunoda K Usuda S Kuramoto J . Comparative single-cell and spatial profiling of anti-SSA-positive and anti-centromere-positive sjögren's disease reveals common and distinct immune activation and fibroblast-mediated inflammation. Nat Commun. (2025) 16:8299. doi: 10.1038/s41467-025-63935-9 40983612 PMC12454658

[20] Shiboski CH Shiboski SC Seror R Criswell LA Labetoulle M Lietman TM . 2016 American College of Rheumatology/European League Against Rheumatism classification criteria for primary sjögren's syndrome: a consensus and data-driven methodology involving three international patient cohorts. Ann Rheum Dis. (2017) 76:9–16. doi: 10.1002/art.39859 27789466

[21] Seror R Ravaud P Bowman SJ Baron G Tzioufas A Theander E . EULAR sjogren's syndrome disease activity index: development of a consensus systemic disease activity index for primary sjogren's syndrome. Ann Rheum Dis. (2010) 69:1103–9. doi: 10.1136/ard.2009.110619 19561361 PMC2937022

[22] Rose T Szelinski F Lisney A Reiter K Fleischer SJ Burmester GR . SIGLEC1 is a biomarker of disease activity and indicates extraglandular manifestation in primary sjögren's syndrome. RMD Open. (2016) 2. doi: 10.1136/rmdopen-2016-000292 28123773 PMC5237743

[23] Feng W Beer JC Hao Q Ariyapala IS Sahajan A Komarov A . NULISA: a proteomic liquid biopsy platform with attomolar sensitivity and high multiplexing. Nat Commun. (2023) 14:7238. doi: 10.1038/s41467-023-42834-x 37945559 PMC10636041

[24] Hansen A Gosemann M Pruss A Reiter K Ruzickova S Lipsky PE . Abnormalities in peripheral B cell memory of patients with primary sjögren's syndrome. Arthritis Rheum. (2004) 50:1897–908. doi: 10.1002/art.20276 15188366

[25] Platanias LC . Mechanisms of type-I- and type-II-interferon-mediated signalling. Nat Rev Immunol. (2005) 5:375–86. doi: 10.1038/nri1604 15864272

[26] Forero A Ozarkar S Li H Lee CH Hemann EA Nadjsombati MS . Differential activation of the transcription factor IRF1 underlies the distinct immune responses elicited by type I and type III interferons. Immunity. (2019) 51:451–464.e6. doi: 10.1016/j.immuni.2019.07.007 31471108 PMC7447158

[27] Hedlund M Thorlacius GE Ivanchenko M Ottosson V Kyriakidis N Lagnefeldt L . Type I IFN system activation in newborns exposed to Ro/SSA and La/SSB autoantibodies in utero. RMD Open. (2020) 6. doi: 10.1136/rmdopen-2019-000989 31958275 PMC7046945

[28] Chen X Jiang S Zhou Z Xu X Ying S Du L . Increased expression of interleukin-21-inducible genes in minor salivary glands are associated with primary sjögren's syndrome disease characteristics. Rheumatol (Oxford). (2020). doi: 10.1093/rheumatology/keaa695 33247940

[29] Rivière E Pascaud J Virone A Dupré A Ly B Paoletti A . Interleukin-7/interferon axis drives T cell and salivary gland epithelial cell interactions in sjögren's syndrome. Arthritis Rheumatol. (2021) 73:631–40. 10.1002/art.41558 33058491

[30] Verstappen GM Gao L Pringle S Haacke EA van der Vegt B Liefers SC . The transcriptome of paired major and minor salivary gland tissue in patients with primary sjögren's syndrome. Front Immunol. (2021) 12:681941. doi: 10.3389/fimmu.2021.681941 34295332 PMC8291032

[31] Michalska A Blaszczyk K Wesoly J Bluyssen HAR . A positive feedback amplifier circuit that regulates interferon (IFN)-stimulated gene expression and controls type I and type II IFN responses. Front Immunol. (2018) 9:1135. doi: 10.3389/fimmu.2018.01135 29892288 PMC5985295

[32] Majoros A Platanitis E Kernbauer-Hölzl E Rosebrock F Müller M Decker T . Canonical and non-canonical aspects of JAK-STAT signaling: lessons from interferons for cytokine responses. Front Immunol. (2017) 8:29. doi: 10.3389/fimmu.2017.00029 28184222 PMC5266721

[33] Woo SJ Im J Jeon JH Kang SS Lee MH Yun CH . Induction of BAFF expression by IFN-γ via JAK/STAT signaling pathways in human intestinal epithelial cells. J Leukoc Biol. (2013) 93(3):363 -8. 10.1189/jlb.0412210 23271704

[34] Sjöstrand M Johansson A Aqrawi L Olsson T Wahren-Herlenius M Espinosa A . The expression of BAFF is controlled by IRF transcription factors. J Immunol. (2016) 196:91–6. 10.4049/jimmunol.1501061 PMC4683359 26590315

[35] Ittah M Miceli-Richard C Eric Gottenberg J Lavie F Lazure T Ba N . B cell-activating factor of the tumor necrosis factor family (BAFF) is expressed under stimulation by interferon in salivary gland epithelial cells in primary sjögren’s syndrome. Arthritis Res Ther. (2006) 8:R51. doi: 10.1186/ar1912 16507175 PMC1526588

[36] Ivanchenko M Thorlacius GE Hedlund M Ottosson V Meneghel L Björkander S . Natural killer cells and type II interferon in Ro/SSA and La/SSB autoantibody-exposed newborns at risk of congenital heart block. Ann Rheum Dis. (2021) 80:194–202. doi: 10.1136/annrheumdis-2019-216786 33004330

[37] Munroe ME Lu R Zhao YD Fife DA Robertson JM Guthridge JM . Altered type II interferon precedes autoantibody accrual and elevated type I interferon activity prior to systemic lupus erythematosus classification. Ann Rheum Dis. (2016) 75:2014–21. doi: 10.1136/annrheumdis-2015-208140 27088255 PMC4959992

[38] Frazzei G Landewé RBM Wagenaar C van de Stadt LA van Schaardenburg D Tas SW . Predictors of arthritis development in individuals at risk of rheumatoid arthritis: a 5-year follow-up study from a large cohort. Ann Rheum Dis. (2025) 84:547–53. doi: 10.1016/j.ard.2025.01.042 39956700

[39] Lima J Martins C Leandro MJ Nunes G Sousa MJ Branco JC . Characterization of B cells in healthy pregnant women from late pregnancy to post-partum: a prospective observational study. BMC Pregnancy Childbirth. (2016) 16:139. doi: 10.1186/s12884-016-0927-7 27267973 PMC4895979

[40] Forbes RL Gibson PG Murphy VE Wark PA . Impaired type I and III interferon response to rhinovirus infection during pregnancy and asthma. Thorax. (2012) 67:209–14. doi: 10.1136/thoraxjnl-2011-200708 21917654

[41] Forbes RL Wark PA Murphy VE Gibson PG . Pregnant women have attenuated innate interferon responses to 2009 pandemic influenza A virus subtype H1N1. J Infect Dis. (2012) 206:646–53. doi: 10.1093/infdis/jis377 22693225

